# The hnRNP family: insights into their role in health and disease

**DOI:** 10.1007/s00439-016-1683-5

**Published:** 2016-05-23

**Authors:** Thomas Geuens, Delphine Bouhy, Vincent Timmerman

**Affiliations:** Peripheral Neuropathy Group, VIB Molecular Genetics Department, University of Antwerp-CDE, Parking P4, Building V, Room 1.30, Universiteitsplein 1, 2610 Antwerp, Belgium; Neurogenetics Laboratory, Institute Born Bunge, University of Antwerp, Antwerp, Belgium

## Abstract

Heterogeneous nuclear ribonucleoproteins (hnRNPs) represent a large family of RNA-binding proteins (RBPs) that contribute to multiple aspects of nucleic acid metabolism including alternative splicing, mRNA stabilization, and transcriptional and translational regulation. Many hnRNPs share general features, but differ in domain composition and functional properties. This review will discuss the current knowledge about the different hnRNP family members, focusing on their structural and functional divergence. Additionally, we will highlight their involvement in neurodegenerative diseases and cancer, and the potential to develop RNA-based therapies.

## Introduction

**Fig. 1:**
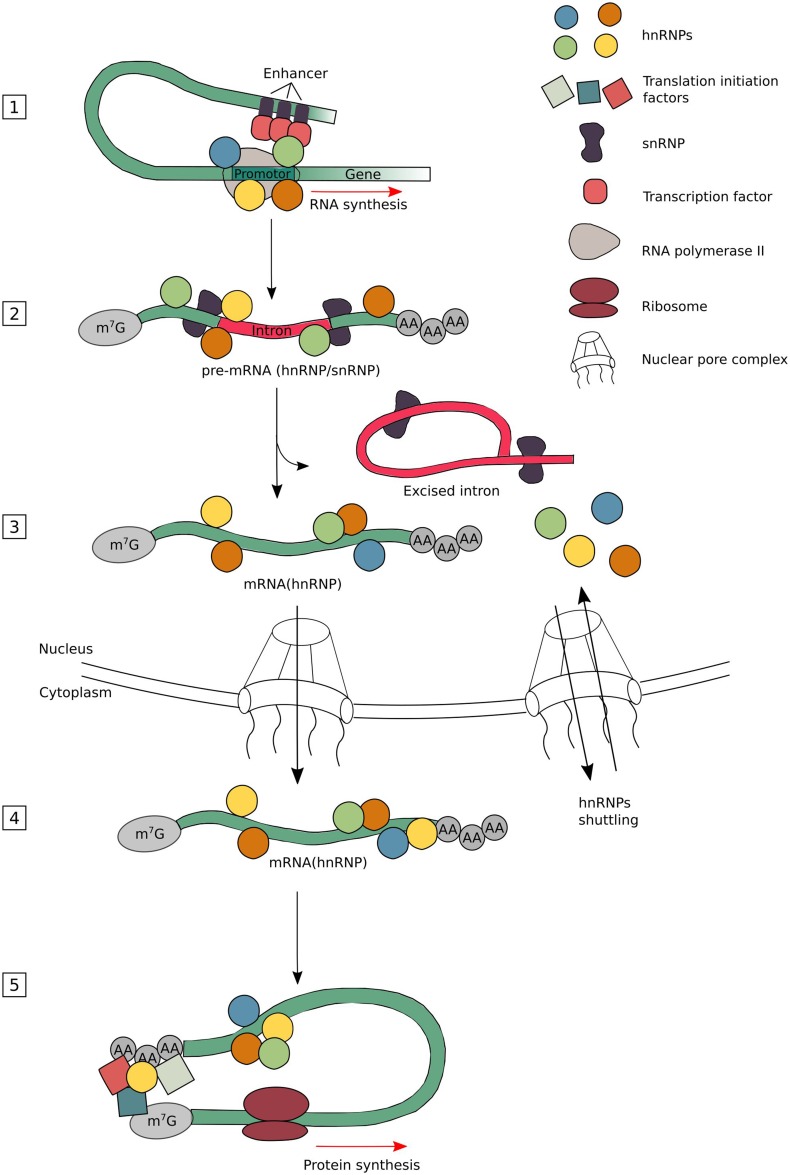
The diverse nuclear and cytoplasmic functions of hnRNPs. The hnRNPs have been found to be involved in different stages of the mRNA metabolism. They constantly undergo a binding and release from their target mRNAs depending on the modification needed. The hnRNPs bind, together with several transcription factors and other RBPs, to promotor and enhancer sequences to direct transcription (**1**). As soon as a part of the newly formed transcript is released by the RNA polymerase II, the hnRNPs and snRNPs bind rapidly to their nascent transcript to stabilize it (**2**). Once the correct RBP complexes are formed, intronic sequences are removed by the spliceosome. Many hnRNPs are known to regulate alternative splicing leading to exon skipping or intron retention (**3**). Mature mRNAs are stabilized by the binding of several types of RBPs, including hnRNPs, which are found necessary for export through the nuclear pore complex (NPC) and its transport through the cytoplasm until translation initiates (**4**). As hnRNPs have the capacity to bind to the 3′- and 5′-UTRs of mRNAs, they can control translational repression or enhancement. Depending on the composition of complexes at the sites where translation initiation factors assemble, the choice to start translation or not will be made (**5**). Please note that not all different hnRNP subgroup members are involved in every nuclear or cytoplasmic function. The different hnRNPs are *color coded*, but do not represent specific hnRNP members

Many ribonucleoproteins (RNPs) assemble on to newly created transcripts in the nucleus of a eukaryotic cell. Among these RNPs are the heterogeneous nuclear ribonucleoproteins (hnRNPs). They assist in controlling the maturation of newly formed heterogeneous nuclear RNAs (hnRNAs/pre-mRNAs) into messenger RNAs (mRNAs), stabilize mRNA during their cellular transport and control their translation. Considering their functional diversity and complexity, hnRNPs act as key proteins in the cellular nucleic acid metabolism (Fig. 1) (Dreyfuss et al. 1993).

The role of hnRNPs in regulating gene expression has gained an increasing interest in disease research. The expression level of hnRNPs is altered in many types of cancer, suggesting their role in tumorigenesis. In addition to cancer, many hnRNPs were also linked to various neurodegenerative diseases, such as spinal muscular atrophy (SMA), amyotrophic lateral sclerosis (ALS), Alzheimer’s disease (AD) and fronto-temporal lobe dementia (FTLD). As neurons are non-dividing cells, therefore needing a tight regulation of mRNA homeostasis, they are highly vulnerable to dysfunction of RNA-binding proteins (RBPs), including the hnRNPs.

**Fig. 2:**
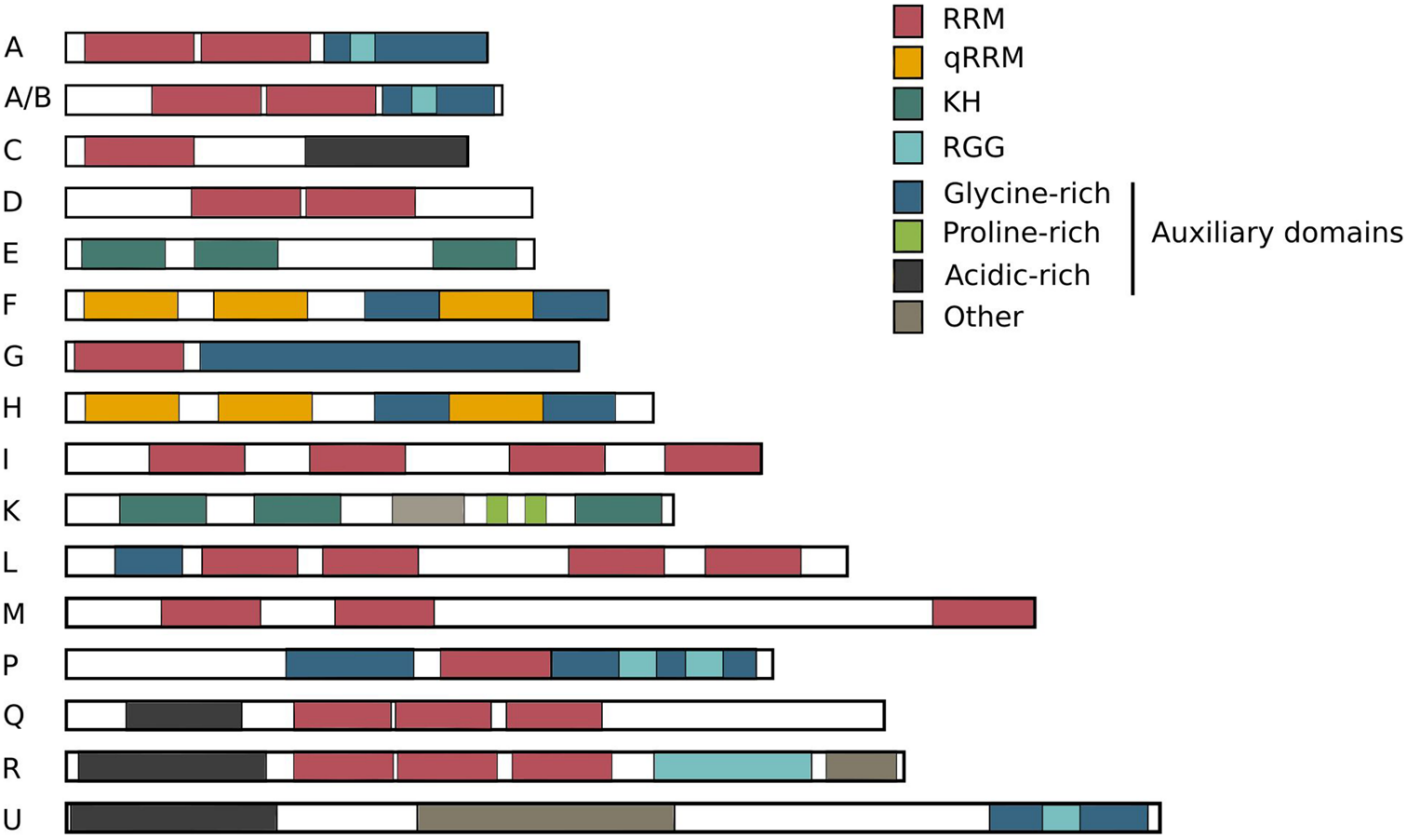
The hnRNP family. The hnRNPs have different molecular weights ranging from 34 to 120 kDa and are named alphabetically from hnRNP A1 to hnRNP U. Many hnRNPs are found to be present in the same complexes, all suggesting that multiple hnRNPs share a common structure and function. As shown in the overview, several structural domains are shared between different family members. The members of the hnRNP family are built up of four unique RNA-binding domains (RBDs). It is obvious that multiple family members carry the same RBDs, partly explaining their shared RNA-binding properties: *RRM* RNA recognition motif, *qRRM* quasi-RNA recognition motif and *KH* K-homology domain, *RGG* RNA-binding domain consisting of Arg-Gly-Gly repeats. The sizes of these 16 common hnRNPs are drawn relative to each other

Because of the growing repertoire of different types of RBPs, it is still unclear how and when they are triggered to interact and functionally complement each other. The hnRNPs were initially categorized according to their RNA-binding domains (RBDs) composition (Dreyfuss et al. 1993), but this classification is not exclusive and many different hnRNPs share common features. This review will focus on the “standard” hnRNPs (Han et al. 2010) (Fig. 2), discuss the overall features of the hnRNP family and highlight their multifunctionality in cellular homeostasis in health and disease.

### The hnRNP family

The 40S core particle was the first mRNA–protein complex, isolated by sucrose density gradients, which comprised hnRNPs A/B and C (Beyer et al. 1977). Later on, several other hnRNPs were identified as RBP complexes via pull-down and UV-cross-linking experiments (van Eekelen et al. 1981). These complexes were shown to be created out of 20 major types of hnRNPs (Table 1) and minor hnRNPs, where the latter ones have been found to be less expressed and do not have hnRNA-binding capacities, but may regulate major hnRNPs (Dreyfuss et al. 1993).

**Table 1:** The hnRNP family presented by their structural and functional characteristics, and their link to diseases

hnRNP	Molecular weight (kDa)	RNA-binding domain	Binding sequence	Function	Link to disease	References
A1	34	2X RRM, Gly rich, RGG	UAGGGA/U	Splicing	ALS/FTLD	(Shan et al. 2003; Liu et al. 2015; Mohagheghi et al. 2015; Park et al. 2015)
				mRNA stability	Cancer	
				Translational regulation		
A2/B1	36/38	2X RRM, Gly rich, RGG	UUAGGG	Splicing	ALS/FTLD	(Hoek et al. 1998; Shan et al. 2003; Qu et al. 2015; Mohagheghi et al. 2015)
				mRNA stability	Alzheimer’s Disease	
					Cancer	
C1/C2	41/43	RRM, acid rich	Poly U	Splicing	Alzheimer’s Disease	(Choi et al. 1986; Lee et al. 2010; Anantha et al. 2013; Borreca et al. 2015)
				Translational regulation	Fragile X Syndrome	
				Transcript sorting	Cancer	
D (AUF1)	44–48	2X RRM	AU rich	mRNA decay	–	(Enokizono et al. 2005; Fialcowitz et al. 2005; Pont et al. 2012)
				Telomere maintenance		
E1/E2/E3/E4	39	3X KH	Poly C	Translational regulation	Cancer	(Ko and Loh 2001; Meng et al. 2007; Waggoner et al. 2009; Chaudhury et al. 2010b)
				Transcriptional regulation		
				mRNA stability		
				Splicing		
F	53	3X qRRM, 2X Gly rich	UUAGG	Splicing	ALS/FTLD	(López de Silanes et al. 2010; Lee et al. 2013)
				Telomere maintenance	Cancer	
G	43	RRM, Gly rich	CC(A/C)	Splicing	SMA	(Moursy et al. 2014)
H	56	3X qRRM, 2X Gly rich	UUAGG	Splicing	ALS/FTLD	(Lee et al. 2013; Gautrey et al. 2015)
					Cancer	
I (PTB1)	59	4X RRM	UCUU(C)	Splicing	–	(Bushell et al. 2006; Söderberg et al. 2007)
				mRNA stability		
				Transcriptional regulation		
K	62	3X KH, other	Poly C	Translational regulation	ALS/FTLD	(Stains et al. 2005; Naarmann et al. 2008; Fukuda et al. 2009b; Cao et al. 2012)
				Transcriptional regulation	Cancer	
				mRNA stability		
				Splicing		
L	68	4X RRM, Gly rich	CA-repeats	Splicing	–	(Söderberg et al. 2007; Melton et al. 2007)
				mRNA stability		
M	77	3X RRM	Poly G/U	Splicing	SMA	(Cho et al. 2014; Xu et al. 2014)
					Cancer	
P (FUS/TLS)	72	2X Gly rich, RRM, 2X RGG	GGUG	Splicing	ALS/FTLD	(Vance et al. 2009; Waibel et al. 2010)
Q1/Q2/Q3	55–70	3X RRM, acid rich	UCUAUC	Splicing	SMA	(Chen et al. 2008; Svitkin et al. 2013)
				Translational regulation		
R	71	3X RRM, acid rich, RGG, other	UCUAUC	Transcriptional regulation	SMA	(Fukuda et al. 2009a; Dombert et al. 2014; Lee et al. 2015)
				Translational regulation		
U	120	Acid rich, other, Gly rich, RGG	GGACUGCRRUCGC	Splicing	–	(Vu et al. 2013; Bi et al. 2013)
				Transcriptional regulation		

The functions of hnRNPs vary according to their cellular localization. The mechanisms that regulate the nucleo-cytoplasmic shuttling are, therefore, of extreme importance. Most of the hnRNP proteins possess a conventional nuclear localization signal (NLS) and are predominantly present in the nucleus during steady state. They are able to translocate in the cytosol upon post-translational stimulation or by the recruitment of other hnRNPs (Fig. 1) (Han et al. 2010). The hnRNP proteins frequently undergo post-translational modifications, leading to changes in biological activity and subcellular localization. Reported post-translational modifications on hnRNPs include methylation, phosphorylation, ubiquitination and sumoylation (Chaudhury et al. 2010a).

### Structure

Four unique RBDs were identified in hnRNP proteins: the RNA recognition motif (RRM), the quasi-RRM, a glycine-rich domain constituting an RGG box and a KH domain (Fig. 2).

The most common RBD is the RNA recognition motif (RRM). Eukaryotic RRMs are structurally characterized by four β-sheets and two α-helices (βαββαβ) together with two conserved RNP1 octameric and RNP2 hexameric sequences that are positioned approximately 30 residues apart from each other (Dreyfuss et al. 1988). It is known that the variable loops, connecting the β-sheets, contribute to its RNA-binding specificity (Görlach et al. 1992).

The RGG box is characterized by contiguous clusters containing aromatic Phe and Tyr residues intercalated between tripeptide repeats of Arg-Gly-Gly amino acids (Kiledjian and Dreyfuss 1992). The glycine-rich domain is often seen as an auxiliary domain, responsible for homologous and heterologous interactions with other hnRNPs (Cartegni et al. 1996).

**Fig. 3:**
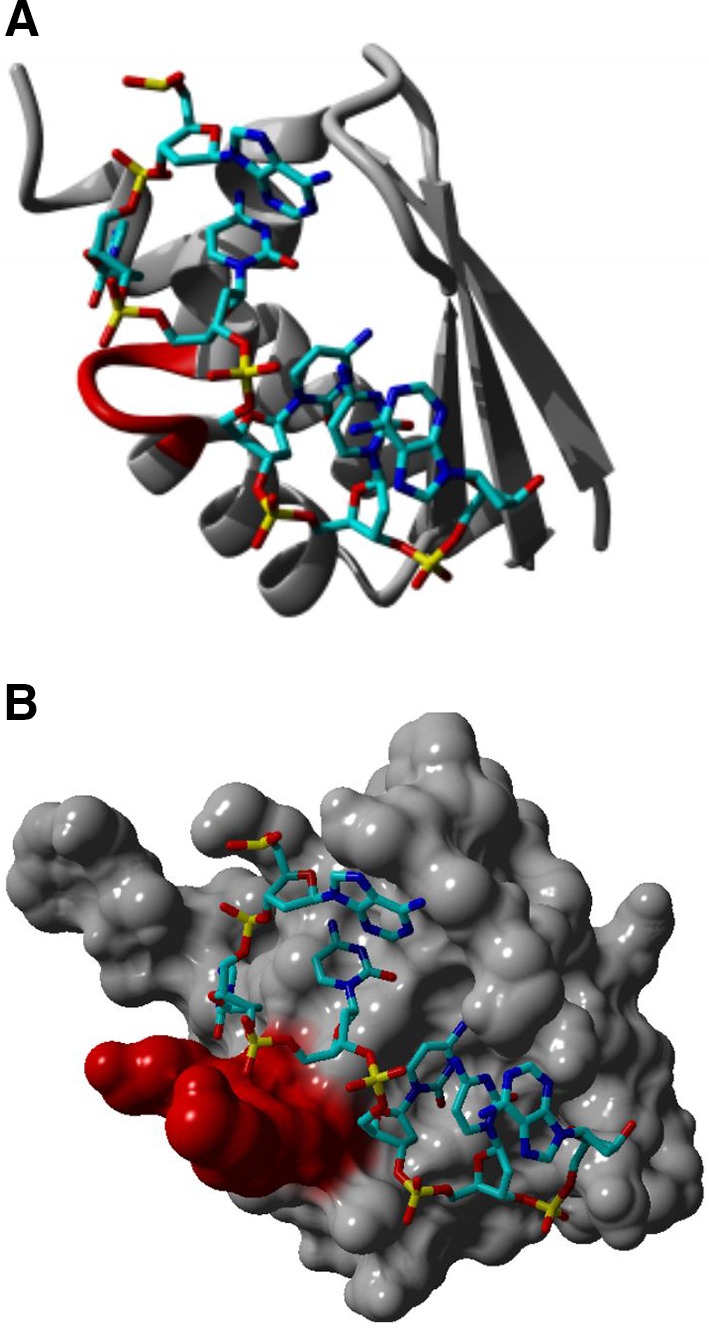
KH–RNA interaction. The KH domain was identified in multiple RNA-binding proteins and characterized by a 45 amino acid repeat that can be split into two groups. The Type I KH domains have a βα extension in their C-terminus, whereas the Type II KH domains have an αβ extension in their N-terminus. The core region of the KH domain is characterized by three-stranded antiparallel β-sheets together with three α-helices (βααββα). It is believed that the nucleotide recognition of the KH domain is determined by a conserved GxxG loop (highlighted in *red*) in the nucleotide stretch that links two α-helices in the KH core (**a**). This leads to the orientation of four nucleotides toward the groove in the protein structure where the nucleotide (backbone colored in *light* and *dark blue*) recognition is mainly determined by hydrophobic interactions and hydrogen bonds. Besides sequence-specific recognition, the overall shape of the KH hydrophobic groove, which is determined by the conformation of multiple side chains, is equally important in KH–RNA recognition (**b**). It was shown by using surface plasmon resonance that the KH domain shows a high affinity for poly(C) repeats, more specifically, the affinity was higher for C-tetrads than for C-triplets. Protein modeling was performed based on the crystal structure of a KH domain bound to a TCCCT DNA sequence, pdb file, 3VKE, and by using the Yasara software (http://www.yasara.org)

The KH domain is originally found in hnRNP K (KH; K-Homology) as triple repeats (Siomi et al. 1993). All KH domains present in hnRNPs are characterized by three-stranded antiparallel β-sheet packed against three α-helices (βααββα). A flexible loop that links two α-helices in the KH core specifically interacts with RNA (Fig. 3) (Jensen et al. 2000). The KH domains are split into two divisions based on their N- or C-terminal extensions. Type I KH domains have a βα extension in their C-terminus, whereas Type II KH domains have an αβ extension in their N-terminus (Grishin 2001).

Next to RBDs, hnRNPs frequently contain auxiliary domains, such as proline-, glycine- or acid-rich domains (Dreyfuss et al. 2002). Not all RBDs are exclusive and the specificity in RNA binding is largely mediated by the 3D structure of the protein where the structural regions around the RBDs fine-tune the RNA–protein interaction.

The modularity created by the combination of these different domains including RNA-binding domains (RBDs) and auxiliary domains (Fig. 2) increases the functional diversity of hnRNPs (Table 1).

## Functional diversity of hnRNPs

### hnRNP A/B

The hnRNPs A/B are divided into four subgroups: hnRNP A1, A2/B1, A3 and A0 for which very little is known. Most studies were performed on hnRNP A1 and A2/B1, which are highly expressed cellular proteins (Dreyfuss et al. 2002) and are found to be involved in mRNA translation (Park et al. 2015) and splicing (Mayeda and Krainer 1992). In addition, hnRNP A2/B1 plays an important role in oligodendrocytic and neuronal mRNA trafficking (Shan et al. 2003). The hnRNP A2/B1 is important for the correct localization of transcripts containing an A2 response element (A2RE) (Shan et al. 2000) or A2RE-like sequences, such as the *myelin basic protein* (MBP) (Makeyev and Liebhaber 2002) or *Ca*^2+^/*calmodulin*-*dependent protein kinase II* (CaMKII), *activity*-*regulated cytoskeleton*-*associated protein* (*Arc*) and *neurogranin* (NRGN) mRNA (Gao et al. 2008). The interactions of hnRNP A2/B1 with A2RE are induced upon elevated cellular Ca^2+^ levels and mediate the dendritic transport of mRNAs (Muslimov et al. 2014). The hnRNP A3 recognize single-stranded telomeric DNA, but due to limited functional studies its cellular function remains largely unknown (Huang et al. 2010).

Recent research identified an additional role of hnRNP A2/B1 in the loading of exosomes. These are plasma membrane-derived extracellular vesicles mediating cell–cell communication and carrying selected proteins, lipids and RNA (Théry et al. 2002). The exact mechanism that regulates the sorting of exosomal RNA remains poorly understood. Recently, hnRNP A2/B1 was shown to bind miRNA and directs it to the exosomes for incorporation (Villarroya-Beltri et al. 2013). This interaction is driven by the recognition of a GGAG motif in the 3′-end of miRNAs and is dependent on the sumoylation of hnRNP A2/B1 (Villarroya-Beltri et al. 2013, 2014). These findings were further supported by the presence of hnRNP A2/B1-miRNA complexes in the exosomes found in human cerebrospinal fluid (Tietje et al. 2014). Similarly, hnRNPA1 and C are able to bind exosomal miRNA; however, no binding motif has been identified so far (Villarroya-Beltri et al. 2013). Exosomes are gaining more attention in therapeutic strategies, as they can be potentially used as vehicles for gene therapy and to deliver vaccines (Lai and Breakefield 2012).

### hnRNP C

The hnRNP C is believed to be the founder of the hnRNP family and was one of the first hnRNPs found to be involved in RNA splicing (Han et al. 2010). Two human spliceoforms have been identified as hnRNP C1 and hnRNP C2, differing from each other by 13 amino acids. The hnRNP C contains only one RNA-binding domain, consisting of an acid-rich auxiliary domain located in the N- or C-terminal RRM (Swanson et al. 1987). Because hnRNP C has only one RBD it has to oligomerize with other hnRNP C monomers to be able to form a strong and specific RNA interaction. This oligomerization capacity is mediated by a leucine zipper motif that lies in the auxiliary domain of hnRNP C (Cieniková et al. 2015). This synergistic interplay between hnRNP C monomers is necessary to form hnRNP C tetramers to measure the length of newly formed transcripts. They bind selectively to unstructured RNA stretches that have a length of more than 200–300 nucleotides, enabling hnRNP C to sort transcripts according to their size (McCloskey et al. 2012). Besides packaging newly formed transcripts, hnRNP C also stimulates the translation of the *c*-*myc* transcription factor (Han et al. 2010).

### hnRNP D (AUF1)

The hnRNP D family, also known as AUF1, comprises four different proteins generated by alternative splicing. These four isoforms constitute two non-identical RRM domains. The hnRNP D forms stable dimers to increase their binding specificity to mRNA targets (Fialcowitz et al. 2005). The four isoforms have a high affinity toward AU-rich and mRNA-destabilizing sequences located in the 3′-UTR of mRNAs (Fialcowitz et al. 2005). Therefore, rapid mRNA decay is most often mediated by the association of hnRNP D (Fialcowitz et al. 2005). Although hnRNP D is mostly known for mRNA decay, it also stimulates the transcription of the *telomerase reverse transcriptase* (TERT) gene, which is required for telomere maintenance (Pont et al. 2012). In addition, this RBP interacts directly with telomeric repeat sequences and further highlights its telomeric function (Enokizono et al. 2005).

### hnRNP E

The hnRNPs E1 and E2, together with hnRNP K, are the only ones containing KH domains to bind RNA (Fig. 3). The hnRNP E1 and E2 are often referred to as poly(C)binding proteins, PCBP1 and PCBP2, respectively, and classified together with hnRNP E3 (PCBP3) and hnRNP E4 (PCBP4) (Leffers et al. 1995) which are exclusively cytoplasmic and therefore not catalogued as hnRNPs (Chkheidze and Liebhaber 2003).

hnRNP E1 and E2 bind to the 3′-UTR of all three neurofilament isoforms (NFL, NFM and NFH), which belong to the family of intermediate filaments and are highly expressed in neurons (Thyagarajan and Szaro 2004). The strength of interaction between NF transcripts and hnRNP E1 and E2 changes during postnatal development in the rat cerebral cortex (Thyagarajan and Szaro 2008). Besides its crucial role in mRNA stabilization, the hnRNP E1 is also known as a modulator of alternative splicing, e.g., of CD44. When hnRNP E1 is phosphorylated by Pak1, it displays an increased nuclear retention, leading to alternative splicing and exon inclusion of a *CD44* mini-gene (Meng et al. 2007). Furthermore, hnRNP E1 is found to negatively control the alternative splicing of *CD44* by forming a complex with THAP11, an important protein for pluripotency and cell growth (Lian et al. 2012). The hnRNP E1 overexpression induces the down-regulation of several variants of the *CD44* transcript (Zhang et al. 2010). Less information is known for the role of hnRNP E1 in the processing of viral RNA, where it interacts with the exon splice site in exon 3 of *HIV1* and thereby alters its protein synthesis (Hadian et al. 2009). Besides their roles in mRNA stability and alternative splicing, hnRNP E1, together with E2, exerts crucial roles in transcriptional and translational regulation. Although hnRNP E1 is classified as an RBP, it also has the capacity to bind to ssDNA. The hnRNP E1 binds, in combination with hnRNP E2 and hnRNP K, to a 26 nucleotide polypyrimidine stretch in the proximal promotor of the *µ-opoid receptor* gene, thereby stimulating its transcription in neuronal cells (Malik et al. 2006), while hnRNP E3 works as a repressor (Choi et al. 2008). Interestingly, hnRNP E1 and E2 depleted cells revealed a marked alteration in the polyadenylation of transcripts. These alterations were seen on transcripts bearing a C-rich motif 30–40 bases, 5′ to their polyadenylation sites (Ji et al. 2013). The best-known and most studied function of hnRNP E1 is its role in translational control. This RBP stimulates translation by interacting with a stem loop in the IRES of its target mRNAs, as seen for poliovirus (Gamarnik and Andino 1997) and *Bag1* mRNA (Pickering et al. 2003). In contrast, it has been proven that hnRNP E1 causes translational repression of the *human papillomavirus type 16* (HPV-16) (Collier et al. 1998), *15*-*lipoxygenase* (15-LOX) (Ostareck et al. 1997), *interleukin*-*like EMT inducer* (ILEI) and *disabled*-*2* (Dab2) (Chaudhury et al. 2010b) mRNAs. Taking together, hnRNP E1 together with hnRNP E2 mediate crucial steps of the RNA metabolism, like the regulation in transcription and translation, mRNA stability and splicing. In contrast to the functional pleiotropy of its close family members, hnRNP E3 is less studied and only reported as a transcriptional repressor.

### hnRNP F/H

Unlike most hnRNPs, hnRNP F and H appear to bind only three consecutive guanines (G-tracts), which are capable of creating G-quadruplexes. The RRMs present in hnRNP F and H are not conserved and are therefore alternatively described as quasi-RRMs (qRRMs) (Dominguez et al. 2010; Samatanga et al. 2013). These qRRMs interact with RNA in a specific way by ‘encaging’ the G-tract RNA sequence (Dominguez et al. 2010). This affinity for repeat sequences allows hnRNP F to recognize the (UUAGG) RNA repeat present in telomeric RNA, suggesting a role for hnRNP F in telomere maintenance (López de Silanes et al. 2010). The best-known functions of hnRNP F and H, in combination with other hnRNPs, is the regulation of alternative splicing (Gautrey et al. 2015).

### hnRNP G (RBMX)

The hnRNP G, alternatively named as RBMX referring to the gene’s position on the X chromosome, is the only hnRNP that can be glycosylated, which is found to be necessary for protein–protein interactions (Soulard et al. 1993). The RBMY is located on the Y chromosome and is thought to have originated from hnRNP G by retrotranspositional events (Elliott et al. 2000) and is only expressed in the testis (Liu et al. 2009). The hnRNP G contains a glycine-rich auxiliary domain that is located in the N- and C-terminal RRM (Han et al. 2010). It is found as a splicing factor, activating the inclusion of exon 7 of the *SMN* gene (Moursy et al. 2014) and inhibiting the splicing of exon 10 of the *tau* gene (Wang et al. 2011). Thus, so far, hnRNP G is only found to be involved in modulating mRNA splicing.

### hnRNP I (PTBP1)/L

The hnRNP I is also known as *polypyrimidine tract*-*binding protein 1* (PTBP1) because it regulates splicing by interacting with polypyrimidine stretches often located at the branch point upstream of exons (Patton et al. 1991). This RNA-binding capacity is mediated by four RRMs found with high sequence similarity in hnRNP L and binding preferentially to CA repeats or CA-rich elements (Han et al. 2010). Surface plasmon resonance spectroscopy underscored the importance of all four RRMs in the contribution to RNA binding and adopting the typical βαββαβ topology (Zhang et al. 2013b). The hnRNP I and L are involved in multiple cellular processes, including mRNA stabilization (Söderberg et al. 2007) and translation (Bushell et al. 2006), but are most probably better known for their activities in pre-mRNA processing.

The hnRNP L is a regulator of inducible exon skipping in *CD45* mRNA upon T cell activation (Melton et al. 2007). Exon binding leads to the repression of strong splice sites and to the enhancement of weak splice sites, possibly by the stabilization of snRNP binding (Motta-Mena et al. 2010). Similarly, the hnRNP I expression level controls alternate 5′ and 3′ splice site usage of its target mRNAs (Hamid and Makeyev 2014) as well as the alternative splicing of the *BCL*-*X* gene, important in cell survival and apoptosis. Both hnRNP I and L interact with each other to suppress exon P3A splicing of the *CHRNA1* gene, encoding one of the subunits of the *nicotinic acetylcholine receptor* (Rahman et al. 2013). Furthermore, both hnRNPs regulate *Cat*-*1* mRNA translation upon starvation conditions (Majumder et al. 2009).

Besides their prominent roles in alternative splicing, the two hnRNPs were recently found to be associated with miRNA-mediated gene regulation. The hnRNP L modulates the expression of the *VEGFA* transcript under hypoxic conditions by competing with the binding of miRNAs in the *VEGFA* 3′-UTR (Jafarifar et al. 2011). Likewise, hnRNP I binds *let*-*7* miRNA and human *Argonaute2*, therefore altering its association to human mRNAs (Engels et al. 2012).

The *neuronal paralog of hnRNP I*/*PTB* (nPTB) has 75 % homology with hnRNP I and has broadly the same effects on the regulation of RNA splicing. However, nPTB and PTB are mostly differentially expressed, leading to distinct outcomes for some neuronal-specific alternative splicing events (Sharma et al. 2005; Zhang et al. 2013a).

### hnRNP K

Similar to hnRNP E1 and E2, hnRNP K has multiple nuclear and cytosolic functions, including the regulation of transcription (Stains et al. 2005), splicing (Cao et al. 2012), mRNA silencing (Fan et al. 2015), mRNA stability (Fukuda et al. 2009b) and translation (Habelhah et al. 2001). What makes hnRNP K different from the other hnRNPs is its functional versatility that is mediated by the capability to interact with multiple proteins through its K interactive region (Bomsztyk et al. 2004). In this way, hnRNP K can be placed at the center of a vast interaction network, enabling it to play multiple roles in diverse cellular processes.

One of the processes in which hnRNP K was newly found to be involved in is the maintenance of ATP levels upon cellular stress, by directly interacting with *RNA binding motif protein 42* (RBM42) mRNA (Fukuda et al. 2009b). The hnRNP K is also integrated in multiple signal transduction pathways by direct binding to the *serotonin transporter* (*SERT*) (Yoon et al. 2013). In addition, hnRNP K is found to interact with *glycogen synthase*-*3β* (*GSK3β*) mRNA, where it regulates various signaling pathways during osteoclast differentiation (Fan et al. 2015).

There is strong evidence for hnRNP K to play an important role in the post-transcriptional regulation of multiple genes involved in the cytoskeletal organization of axons, and hnRNP K is therefore found to be essential for axonogenesis (Liu and Szaro 2011). This regulation is directed by the phosphorylation of hnRNP K by the c-Jun N-terminal kinase (JNK) (Hutchins and Szaro 2013). Corroborating this, the knockdown of hnRNP K results in the failure to create axons in developing *Xenopus* embryos (Liu et al. 2008). In addition, a mouse model harboring an *hnRNP K* knockout allele results in reduced survival and increased tumor formation (Gallardo et al. 2015). The importance of hnRNP K during embryonal development was supported by the appreciation of high protein expression levels of hnRNP K in the central and peripheral nervous system (Blanchette et al. 2006).

### hnRNP M/Q

The hnRNP M and Q are classified together, as these two RBPs have shared RNA-binding properties.

The hnRNP M is an abundant protein composed of three RRMs and is mostly present in the nucleus where it acts as a component of the spliceosome complex (Llères et al. 2010). This association is further confirmed by the identification of hnRNP M in nuclear speckles that are enriched with splicing factors (Marko et al. 2010). Since the first report where hnRNP M was identified as a splicing regulator for *fibroblast growth factor receptor 2* (*FGFR2*) (Hovhannisyan and Carstens 2007), many reports followed and identified new targets: *carcinoembryonic antigen*-*related cell adhesion molecule*-*1* (*CEACAM1*) (Dery et al. 2011), *dopamine D2 receptor* (*D2M*) (Park et al. 2011) and *survival motor neuron 1 and 2* (*SMN1* and *SMN2*) (Cho et al. 2014).

The hnRNP Q (also known as SYNCRIP) has three isoforms called hnRNP Q1-Q3. They are all created by alternative splicing events. The N-terminus has three RRMs and an auxiliary domain composed of acidic residues. Although the exact molecular function of hnRNP Q is not yet fully understood, many steps of mRNA maturation have been found to be associated (Mourelatos et al. 2001). Recent studies in mice show a possible novel role for hnRNP Q2 by binding to the poly(A)repeats of mRNAs and therefore competing with the poly(A)binding protein (PABP) (Svitkin et al. 2013). Interestingly, hnRNP Q contributes to the dendritic development and focal adhesion formation in neurons (Xing et al. 2012). Knockdown experiments in cortical neurons of mice show an increased axonal and neurite length (Williams et al. 2015).

### hnRNP P2 (FUS/TLS)

The hnRNP P2 is better known as the *RNA binding protein fused in sarcoma/translated in liposarcoma* (*FUS/TLS*), which is mutated in familial and sporadic ALS patients (Vance et al. 2009). FUS/TLS contains one RRM and two glycine-rich domains, mediating its RNA-binding capacities. FUS/TLS contains an NLS and is predominantly located in the nucleus. The main part of the reported mutations are located in the NLS of the C-terminus, resulting in an increased cytoplasmic retention (Waibel et al. 2010). The reported function of FUS/TLS will be further discussed in Box2—Role of hnRNPs in neurodegenerative diseases.

### hnRNP R/U

The hnRNP R contains three RRMs and one RGG box, allowing specific interactions with its mRNA targets (Han et al. 2010). The hnRNP enhances *c*-*fos* transcription, by forming a complex with PC4 and Mediator (Fukuda et al. 2009a). The presence of the above-mentioned RBDs suggests additional post-transcriptional roles. More importantly, hnRNP R is also found in the cytosol where it regulates cap-independent translation by binding to the IRES of target mRNAs (Lee et al. 2015).

The hnRNP U is the largest hnRNP family member and is mainly present in the nucleus where it is able to bind pre-mRNA and ssDNA. It contains an N-terminus rich in acidic residues and a glycine-rich C-terminus, and within the middle of the protein a glutamine stretch together with an NLS sequence. It is believed that the RNA-binding capacities of hnRNP U are mediated by an RGG box in the glycine-rich C-terminus (Han et al. 2010). Because of its predominant nuclear localization, it is mainly involved in transcription (Bi et al. 2013) and alternative splicing (Vu et al. 2013). Interestingly, hnRNP U is needed for the accumulation of the lncRNA *Xist* on chromosome X, therefore epigenetically inactivating one of the two female X chromosomes to equalize gene expression with male mammals (Hasegawa et al. 2010).

## Conclusions

The hnRNPs are RBPs responsible for packing and stabilizing freshly transcribed pre-mRNAs. This early assembly step facilitates the removal of introns and alternative exons, leading to fully mature mRNAs ready to be exported out of the nucleus to translation-active sites in the cytoplasm. More recent studies have shown that hnRNPs have the potential to play diverse, but important roles in maintaining mRNA homeostasis in the nucleus and cytoplasm. The functional versatility within the hnRNP family can arise from the ability of hnRNPs to form complexes with other hnRNP members and the potential to generate multiple alternatively spliced isoforms. Additional auxiliary domains, such as acid-, proline- and glycine-rich domains found in some hnRNPs to date, indicate that hnRNPs can be regulated in alternative ways. It is obvious that the insights into the functional regulation of hnRNPs is still limited and will definitely be explored in the following years. This additional knowledge will be beneficial for the development of new therapeutics as the number of hnRNPs involved in cancer and neurodegenerative disease is rapidly increasing.

To deeply investigate these shortcomings, it is of outmost importance to have more structural information of the protein. The limited number of hnRNP structures currently available already points out the difficulties in studying these proteins at a 3D level. Mainly, the highly variable loops/regions that reside in between the conserved RNA-binding domains put extra challenges on these studies to succeed. Another question that needs to be answered is how the hnRNPs can specifically bind to their mRNA targets. To date, only a very small number of these protein–RNA complexes were resolved with high resolution using X-ray crystallography (Chaudhury et al. 2010a).

Besides the importance of flexible loops in the recognition of its targets, the secondary structure of RNA is of crucial importance (Wu et al. 2004). To understand the complexity of hnRNP binding to mRNA targets, there is a need for the determination of a 3D structure on the protein level, but preferentially also on the protein–RNA level. Bioinformatic tools will be useful to predict these protein–RNA interactions and to help resolve its 3D structure.

Of equal importance will be the identification of their mRNA targets. Already, efforts have been made for some hnRNPs, like for example hnRNP L, where cross-linked immunoprecipitation (CLIP) experiments shed light on the differential splicing regulation (Rossbach et al. 2014). As many hnRNPs have common RNA-binding properties, these generated data will contain additional information about the shared mRNA targets between hnRNPs. Additionally, the identification of various complexes formed between the hnRNP members will help us to better understand their complementary functions.

It has become clear that hnRNPs are crucial players in the development of cancer and neurodegenerative disease. Looking at the important cellular functions of this group of RBPs, it would not be surprising to find them associated with other diseases in the near future. The rise in bio-informatics, improved biochemical approaches and in vivo models significantly increased our knowledge of RBPs in human diseases. However, the underlying pathomechanisms of many hnRNPs remain to be elucidated by studying their common and more specific functions related to disease. Nowadays, extensive research is performed on modulating the function of these RBPs to restore the disease phenotype. An example in cancer research is the recent unraveling of the epithelial–mesenchymal transition pathway, which was shown to be regulated by hnRNP E1 (Hussey et al. 2011). Looking at neurodegenerative diseases, it was clearly shown that RNA-mediated toxicity is a hallmark of many affected neurons. For example, the C9orf72 repeat expansion in ALS/FTLD patients are causative for RNA foci entrapping a huge collection of RBPs, which are, as a consequence, not functional (Mori et al. 2013). Future RNA-based therapies will focus on the attenuation of this RNA toxicity, achieved by antibodies targeting the foci for degradation or by small molecules inhibiting *repeat*-*associated non*-*ATG* (*RAN*) translation. An alternative approach used in RNA-based therapy is to prevent the formation of toxic RNA foci. A critical step is to reduce the expression level of the disease-propagating protein, which can be done by protein translation arrest or mRNA degradation. Antisense oligonucleotide drugs (AODs) can directly bind to mRNA or interfere with the binding of miRNAs to their target. Promising research done on transgenic C9orf72 mice already showed that the level of mutant pathogenic mRNAs could be lowered by the administration of AODs, therefore reducing RNA toxicity and disease-associated symptoms (Lagier-Tourenne et al. 2013; Riboldi et al. 2014). This therapeutic strategy resulted in a first attempt to patent the use of AODs as a novel treatment for ALS/FTLD patients (Evers et al. 2015). Recent findings reporting a translational dysregulation in neurodegenerative diseases, caused by mutations in novel or known hnRNPs or by mutations in miRNAs, indicate the vulnerability of this regulation system in neurons. Therefore, characterizing hnRNPs on a structural and functional level will be a crucial challenge.

### BOX 1: role of hnRNPs in cancer

**Fig. 4:**
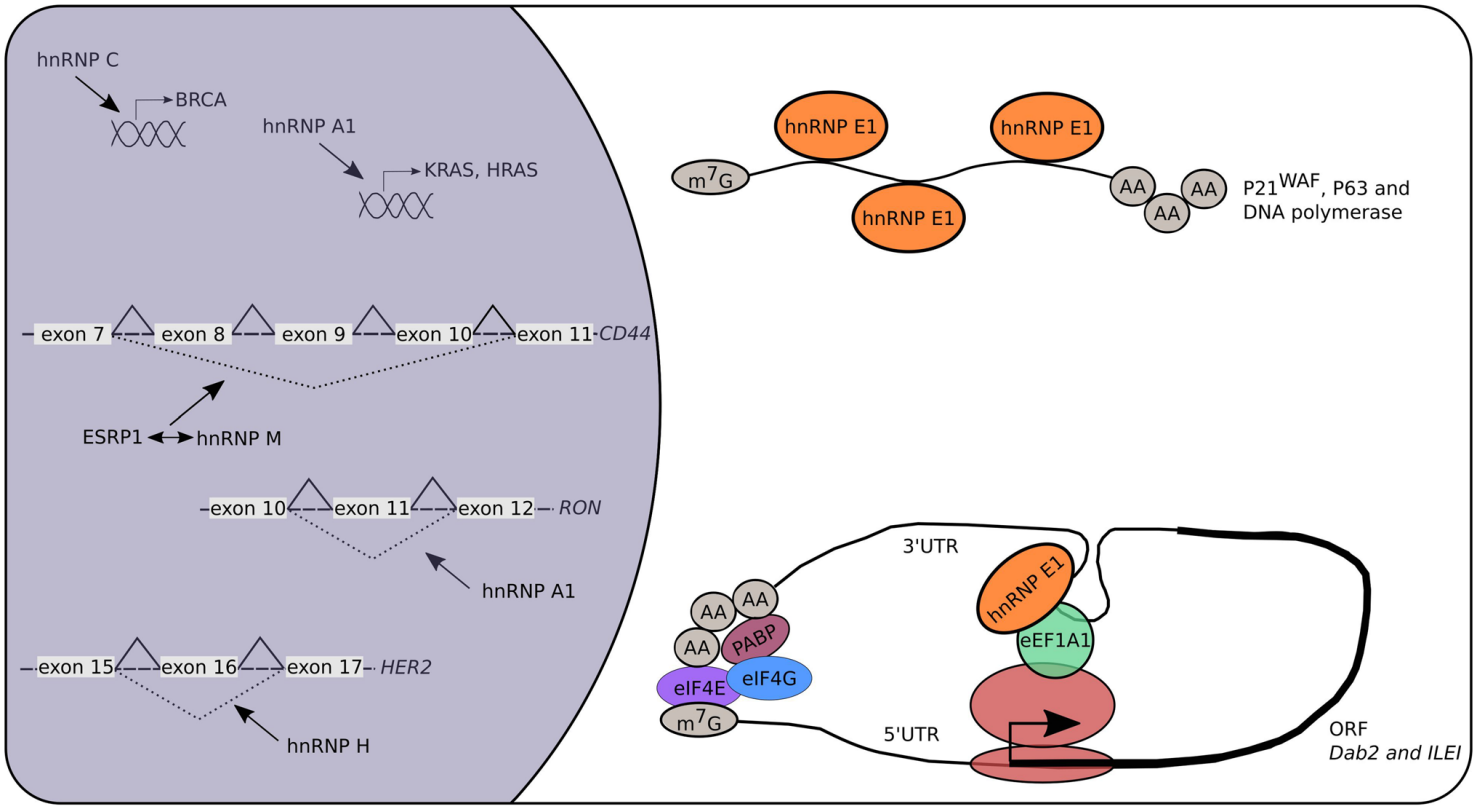
The role of hnRNPs in cancer. Multiple hnRNPs are linked to cancer and metastasis. Many of them act on the level of alternative splicing leading to truncated proteins because of exon skipping. This modulation can be direct (e.g., hnRNP A1 and H) or indirect (e.g., hnRNP M) by competing with other RBPs like ESRP1. Additionally, hnRNP A1 and C are known transcriptional regulators acting on the promotor of *KRAS, HRAS* and *BRCA*, respectively. Specific cytosolic functions of hnRNP E1 are linked to the initiation and progression of cancers. More specifically, hnRNP E1 controls the mRNA stability and therefore mRNA half-life of specific genes (p21^WAF^, p63, DNA polymerase η) linked to cancer development. Recently, the discovery that hnRNP E1 is a key player in the TGF-β-mediated EMT resulted in deciphering RBP components involved in the translational control of known oncogenes like *Dab*-*2* and *ILEI*

Many RBPs were identified to be involved in cancer and metastasis (Fig. 4). Because hnRNPs are important for gene expression regulation and show functional overlap; it is not surprising that many of the hnRNPs are keystones in tumor development. The hnRNP A1 is a major player and has been associated with different types of cancer and metastasis. Its expression is dramatically increased in lung cancer samples and is associated with tumor proliferation (Liu et al. 2015). In addition, the knockdown of hnRNP A2/B1 in MDA-MB-231 cells decreases cell invasion and thereby the proliferative effect of tumor cells (Loh et al. 2015). Therefore, nanoparticle-aptamers were developed that recognize hnRNP A2/B1 in multiple tumor cells (Li et al. 2015). They consist of small oligomeric structures conjugated with nanoparticles, enabling them to easily reach cellular targets and to mediate a translational repression. Several oncogenes have been identified as being direct targets of hnRNP A1, such as *Kirsten rat sarcoma viral oncogene homolog* (*KRAS*), *Harvey rat sarcoma viral oncogene homolog* (*HRAS*) and a splice variant of *Recepteur d’Origine Nantais* (*ΔRON*), and can therefore control tumorigenesis and metastasis indirectly (Jean-Philippe et al. 2013). Another oncogene named *breast cancer* (*BRCA*) was found to be regulated by hnRNP C. It was suggested that hnRNP C also acts as a key regulator controlling the metastatic potential of glioblastoma cells (Anantha et al. 2013).

Alternative splicing can increase the proteomic diversity, which is necessary for the flexibility of the cell to respond to various conditions. It is therefore of outmost importance that the production of alternative transcripts is highly regulated. In case of dysregulation, splice variants can lead to aberrant protein isoforms having altered functions. Recently, hnRNP H was identified as one of the first splicing factors leading to the production of *HER2* splice variants, being important in the HER2 signaling pathway in cancer patients (Gautrey et al. 2015). Similarly, hnRNP M is involved in breast cancer where it activates the switch of alternative splicing, thereby precisely controlling *CD44* splice isoforms, leading to epithelial–mesenchymal transition (EMT) (Xu et al. 2014).

Probably, the best-studied hnRNP in cancer research is hnRNP E1. Its role in gene expression regulation, either on transcriptional or translational level, makes hnRNP E1 a key regulator of many cellular proteins, including proteins whose under- or overexpression can lead to disease. For example, the cyclin-dependent kinase inhibitor p21^WAF^ is a key mediator of the p53-dependent cell cycle arrest, playing a role in tumor suppression (Oren and Rotter 2010). The hnRNP E1 together with hnRNP E2 control the *p21*^*WAF*^ mRNA half-life and therefore regulate the cell cycle via a p53-independent mechanism (Waggoner et al. 2009). Additionally, hnRNP E1 increases the mRNA stability of *DNA polymerase η*, which is a target of p53 tumor suppression (Ren et al. 2014). The p63, another transcription factor and a p53 family protein, was also identified to be a target of hnRNP E1 mRNA stabilization (Cho et al. 2013). Another example where hnRNP E1 translationally represses genes involved in tumorigenesis is its involvement in EMT (Chaudhury et al. 2010b). Many hnRNP E1 targets were identified to be associated with EMT, including *Dab2* and *ILEI* (Hussey et al. 2011). Supporting these findings, knockdown of hnRNP E1 results in the formation of tumors in mice after subcutaneous injection of NMuMG cells (Hussey et al. 2011).

### BOX 2: role of hnRNPs in neurodegenerative diseases

**Fig. 5:**
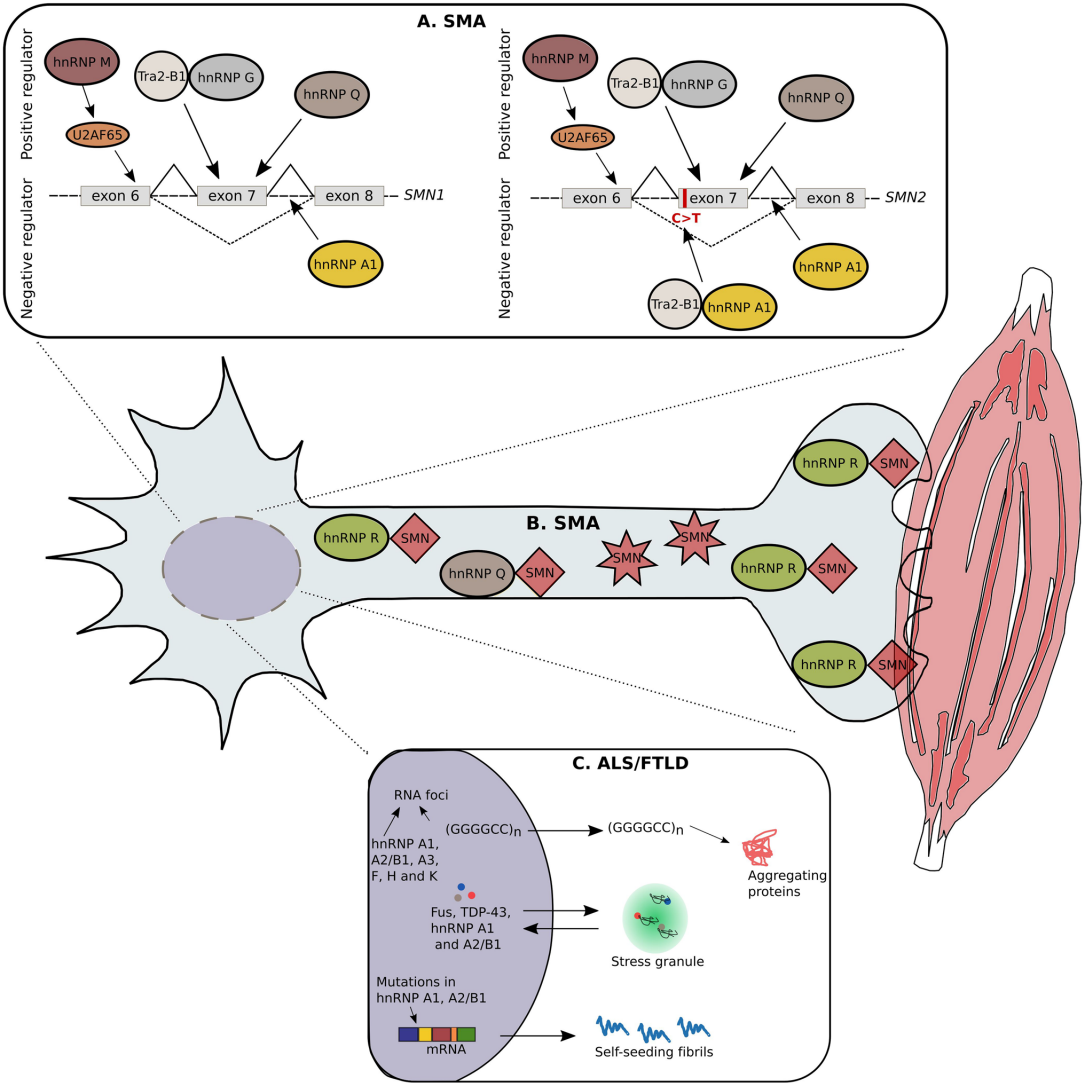
hnRNPs involved in SMA and ALS/FTLD. Due to extensive research in the last years, numerous hnRNPs are linked with SMA and ALS/FTLD. A single nucleotide substitution in the 5′-end of exon 7 in the *SMN2* gene leads to exon skipping and consequently to a non-functional SMN protein. The hnRNP G can form a complex with the positive modifier, Tra2-β1, resulting in the retention of exon 7. In addition, hnRNP Q can bind to the single nucleotide substitution, thereby avoiding exon 7 skipping. The hnRNP M targets a splicing enhancer on exon 7 through the recruitment of U2AF65, leading to the production of a full-length transcript. In contrast, hnRNP A1 is found to be a negative regulator by binding to the splicing silencer, created by the single nucleotide substitution in *SMN2*. In addition, hnRNP A1 can bind to regions located in the introns of *SMN1/2* (**a**). Besides being involved in alternative splicing, the hnRNP Q forms protein interactions with wild-type SMN, but is unable to bind to the truncated form. Similarly, the hnRNP R interacts with SMN in the cytosol of motor neurons; more specifically this interaction is present in the presynapses of neuromuscular junctions (**b**). C9orf72 repeat expansions causing ALS/FTLD leads to the formation of RNA foci containing hnRNP A1, A2/B1, A3, F, H and K suggesting RNA toxicity via the sequestration of RBPs as a possible pathomechanism. Furthermore, hnRNP A1 and A2/B1 are sequestered in cytosolic stress granules together with other RBPs causative for ALS/FTLD, such as FUS and TDP-43. Mutations found in the prion-like domain of hnRNP A1 and A2/B1 are causative for ALS/FTLD. In normal conditions, these hnRNPs have an intrinsic tendency to self-aggregate, because of their prion-like domains, but this tendency is abnormally increased because of the disease-causing mutations (**c**)

Axons in the peripheral nervous system can be as long as 1 m and are therefore dependent on local translation. The regulation of translation at the presynaptic sites is of extreme importance as well as the transport of stabilized mRNAs along the axonal cytoskeleton to these local translation hubs. Not surprisingly, hnRNPs, together with other RBPs, are crucial for the coordination of these processes. Perturbations in their functions and the control of target mRNAs can lead to multiple neurological diseases, such as ALS/FTLD, SMA and AD (Fig. 5) (Bekenstein and Soreq 2013).

Mutations found in the prion-like domain located in hnRNP A1 and A2/B1 are causative for ALS/FTLD. In normal conditions, these hnRNPs have an intrinsic tendency to self-aggregate, because of their prion-like domains, but this tendency is abnormally increased by the disease-causing mutations (Kim et al. 2013). Although mutations in hnRNP A1 and A2/B1 further support the idea of an altered RNA metabolism as the underlying pathomechanism for ALS/FTLD, it was found that these mutations are still a rare cause of the disease because of their low frequency (Le Ber et al. 2014). In addition, different genetic causes, like mutations in the Tar DNA-binding protein 43 (TDP-43) and *C9orf72* repeat expansions, were also found in ALS/FTLD patients. Motor neurons derived from induced pluripotent stem cells (iPSCs), originating from these ALS/FTLD patients, clearly showed the presence of hnRNP A1 and A2/B1 in RNA foci, suggesting RNA toxicity via the sequestration of RBPs as a possible pathomechanism (Mohagheghi et al. 2015). More specifically, other hnRNPs can also be sequestered in these RNA foci as it was found for hnRNP A3, F, H and K in the spinal cord of ALS/FTLD patients (Cooper-Knock et al. 2015). Another hnRNP mutated in ALS patients is hnRNP P2, better known as FUS/TLS. The majority of the reported mutations are clustered in the C-terminal NLS leading to an increased cytoplasmic retention (Waibel et al. 2010).

Mutations in the *survival motor neuron1* (*SMN1*) gene encoding for an RNA-binding protein are causative for SMA. A paralog gene, *SMN2*, differs from *SMN1* by a C/T transition in exon 7, which results in a lower expression of full-length SMN protein that cannot rescue the loss of *SMN1* expression. A strategy used to treat SMA patients is to promote exon 7 retention in *SMN2* (Porensky and Burghes 2013). Although a common pathomechanism between ALS/FTLD and SMA remains unclear, it was shown that TDP-43 and FUS/TLS co-localize in nuclear speckles together with SMN. The formation of such a complex suggests that all three proteins function in spliceosome maintenance. Interestingly, the spliceosomal machinery is affected in both neurological diseases (Tsuiji et al. 2013). Currently, five hnRNPs (A1, G, M, Q and R) are linked to SMA (Kashima et al. 2007; Chen et al. 2008; Cho et al. 2014; Dombert et al. 2014; Moursy et al. 2014) and are extensively discussed in Fig. 5.

The hnRNP C is linked to Alzheimer’s disease (AD) characterized by amyloid-b plaques formed by the amyloid-beta protein, which is a cleavage product of APP. The translational regulation of *APP* mRNA is therefore of crucial importance and its sequence is a target of both fragile X mental retardation protein (FMRP) and hnRNP C. Both RBPs act competitively and influence APP translation in an opposing way (Borreca et al. 2015).

## Abbreviations

A2RE: A2 response element
AD: Alzheimer’s disease
ALS: Amyotrophic lateral sclerosis
APP: Amyloid precursor protein
Dab2: Disabled-2
EMT: Epithelial–mesenchymal transition
FTLD: Fronto-temporal lobe dementia
hnRNP: Heterogeneous nuclear ribonucleoprotein
HPV-16: Human papillomavirus type 16
ILEI: Interleukin-like EMT inducer
IRES: Internal ribosome entry site
KH: K-Homology
MBP: Myelin basic protein
NF: Neurofilament
NLS: Nuclear localization signal
PTBP1: Polypyrimidine tract-binding protein 1
qRRM: Quasi RNA recognition motif
RBD: RNA-binding domain
RBP: RNA-binding protein
RGG box: Arg-Gly-Gly box
RNP: Ribonucleoprotein
RRM: RNA recognition motif
SMA: Spinal muscular atrophy
SMN: Survival motor neuron
snRNP: Small nuclear ribonucleoprotein
TGF-β: Transforming growth factor-beta
UTR: Untranslated region
15-LOX: 15-Lipoxygenase
